# Impact of dietary fiber intake on glycemic control, cardiovascular risk factors and chronic kidney disease in Japanese patients with type 2 diabetes mellitus: the Fukuoka Diabetes Registry

**DOI:** 10.1186/1475-2891-12-159

**Published:** 2013-12-11

**Authors:** Hiroki Fujii, Masanori Iwase, Toshiaki Ohkuma, Shinako Ogata-Kaizu, Hitoshi Ide, Yohei Kikuchi, Yasuhiro Idewaki, Tamaki Joudai, Yoichiro Hirakawa, Kazuhiro Uchida, Satoshi Sasaki, Udai Nakamura, Takanari Kitazono

**Affiliations:** 1 Department of Medicine and Clinical Science, Graduate School of Medical Sciences, Kyushu University, Maidashi 3-1-1, Higashi-ku, Fukuoka 812-8582, Japan; 2 Diabetes Centre, Hakujyuji Hospital, Fukuoka, Japan; 3 Department of Environmental Medicine, Graduate School of Medical Sciences, Kyushu University, Fukuoka, Japan; 4 Department of Health Promotion, School of Health and Nutrition Science, Nakamura, Gakuen University, Fukuoka, Japan; 5 Department of Social and Preventive Epidemiology, School of Public Health, The University of Tokyo, Tokyo, Japan

**Keywords:** Adiponectin, Albuminuria, Chronic kidney disease, Diabetes mellitus, Dietary fiber, Homeostasis model assessment, Hypertension, Inflammation, Insulin resistance, Metabolic syndrome

## Abstract

**Background:**

Dietary fiber is beneficial for the treatment of type 2 diabetes mellitus, although it is consumed differently in ethnic foods around the world. We investigated the association between dietary fiber intake and obesity, glycemic control, cardiovascular risk factors and chronic kidney disease in Japanese type 2 diabetic patients.

**Methods:**

A total of 4,399 patients were assessed for dietary fiber intake using a brief self-administered diet history questionnaire. The associations between dietary fiber intake and various cardiovascular risk factors were investigated cross-sectionally.

**Results:**

Body mass index, fasting plasma glucose, HbA1c, triglyceride and high-sensitivity C-reactive protein negatively associated with dietary fiber intake after adjusting for age, sex, duration of diabetes, current smoking, current drinking, total energy intake, fat intake, saturated fatty acid intake, leisure-time physical activity and use of oral hypoglycemic agents or insulin. The homeostasis model assessment insulin sensitivity and HDL cholesterol positively associated with dietary fiber intake. Dietary fiber intake was associated with reduced prevalence of abdominal obesity, hypertension and metabolic syndrome after multivariate adjustments including obesity. Furthermore, dietary fiber intake was associated with lower prevalence of albuminuria, low estimated glomerular filtration rate and chronic kidney disease after multivariate adjustments including protein intake. Additional adjustments for obesity, hypertension or metabolic syndrome did not change these associations.

**Conclusion:**

We demonstrated that increased dietary fiber intake was associated with better glycemic control and more favorable cardiovascular disease risk factors including chronic kidney disease in Japanese type 2 diabetic patients. Diabetic patients should be encouraged to consume more dietary fiber in daily life.

## Background

Diet rich in dietary fiber is beneficial for the treatment of type 2 diabetes mellitus [1], as dietary fiber ameliorates postprandial hyperglycemia by delaying digestion and absorption of carbohydrates and enhances satiety, which leads to a reduction in body weight [2]. In insulin-resistant subjects, dietary fiber may enhance peripheral insulin sensitivity possibly via short-chain fatty acids produced by fermentation of fiber in the intestines [3-5]. The hypoglycemic actions of dietary fiber in type 2 diabetic patients have been investigated by conducting interventions with high fiber diets or supplements [2]. In addition, an epidemiologic study [6] recently reported that HbA1c was significantly lower in type 2 diabetic patients with high fiber intake than in those with low fiber intake among 934 Chinese subjects who ate foods containing larger amounts of fiber than the Western diet [7].

As for cardiovascular disease (CVD) risk factors in type 2 diabetic patients, the effects of dietary fiber were not fully explored. Soluble fiber forms gels in the gastrointestinal tract, and may decrease the absorption of glucose and cholesterol from the intestinal lumen [8]. High fiber diet improved diabetic dyslipidemia in some studies [9], and a low fiber intake was associated with metabolic syndrome in Brazilian type 2 diabetic patients [10]. Although the consumption of whole grains rich in insoluble fiber was reported not to be associated with improvements in glycemic control [9,11], it suppressed low-grade systemic inflammation [12] and was inversely associated with all-cause and CVD-specific mortality among diabetic females in the Nurses’ Health Study [13]. Recently, it was reported that increased dietary fiber, especially soluble fiber intake was associated with reduced all-cause and CVD-specific mortality in type 1 diabetic patients [14]. However, a recent review reported that adding fiber supplements in moderate amounts (4–19 g) to daily diet leads to little improvement in glycemic or CVD risk markers, although the effects of dietary fiber were investigated mostly in subjects consuming Western diet [9]. Dietary fiber is consumed differently in ethnic foods around the world, and the protective effects of dietary fiber on the development of diabetes differed by ethnic group according to consumed foods [15]. Japanese foods consist of dietary fiber primarily in the form of vegetables including seaweed, and contain smaller amounts of fiber than Western diet, of which the main source of fiber is whole grains [7,16]. It has been reported that increased intake of dietary fiber is associated with reduced mortality from CVD in the Japanese general population [17,18], although the effects of dietary fiber intake have not been investigated in diabetic patients. In the present study, we investigated the associations of dietary fiber intake with glycemic control and CVD risk factors, i.e., metabolic syndrome, LDL cholesterol, low-grade inflammation and chronic kidney disease (CKD) in Japanese type 2 diabetic patients. This cross-sectional study suggested the beneficial effects of dietary fiber on glycemia and a wide range of CVD risk factors including CKD.

## Methods

### Subjects

The Fukuoka Diabetes Registry is a multicenter prospective study designed to investigate the effects of modern treatment on the prognosis of diabetic patients attending teaching hospitals certified by the Japan Diabetes Society or certified diabetes clinics in Fukuoka Prefecture, Japan (UMIN Clinical Trial Registry 000002627) [19]. A total of 5,131 diabetic patients 20 years of age or older were registered between April 2008 and October 2010. After excluding 261 subjects with type 1 diabetes mellitus, 468 subjects who had already eaten breakfast and three subjects who reported consuming less than 500 kcal in a dietary survey, the remaining 4,399 subjects (2,493 males, 1,906 females) were enrolled in this cross-sectional study. The exclusion criteria were: 1) patients with drug-induced diabetes and those receiving steroid treatment, 2) patients under renal replacement therapy and 3) patients with serious diseases other than diabetes, such as advanced malignancy, decompensated liver cirrhosis, etc. and 4) patients unable to visit diabetologists regularly. This study was conducted with the approval of the Kyushu University Institutional Review Board, and written informed consent was obtained from all participants.

### Dietary assessment

The dietary survey was conducted using a brief self-administered diet history questionnaire (BDHQ) to assess the subjects’ dietary intake during the preceding month. The BDHQ includes 58 foods and beverage items. The subjects indicated their mean frequency of consumption in terms of the specified serving size by checking one of seven frequency categories ranging from “almost never” to “two or more times a day” [20]. The dietary intake estimates for total energy and several nutrients, including dietary fiber, were calculated using an ad hoc algorithm developed for the BDHQ based on the Standard Tables of Food Composition in Japan [21]. Validation of ranking energy-adjusted fiber intake has been previously studied in an adult Japanese population [22]. The correlation coefficient of dietary fiber intake between semi-weighed dietary records for 16 days and BDHQ was 0.66 in females (n = 92) and 0.70 in males (n = 92), respectively.

### Clinical evaluation

Participants completed a self-administered questionnaire concerning the duration of diabetes mellitus, alcohol intake, smoking habits and physical activity. Body mass index (BMI) was calculated from each subject’s height and weight, and obesity was defined as BMI of ≥25 kg/m^2^ according to Japan Society for the Study of Obesity [23]. Waist circumference at the umbilical level was measured by a trained staff member with the subject in the standing position, and blood pressure was measured with the subject in the sitting position. The subjects’ medical records were reviewed for oral hypoglycemic agent and insulin use. Leisure time physical activity information was obtained using a self-reported questionnaire, and metabolic equivalent (met) hours per week was calculated using Ainsworth’s methods [24].

### Laboratory measurements

Blood was collected via venipuncture. Spot urine samples were obtained, and the assessments were performed at one central laboratory. HbA1c was determined using high-performance liquid chromatography (Tosoh Corp., Tokyo, Japan), plasma glucose by glucose oxidase method, serum C-peptide by chemiluminescent immunoassay (Kyowa Medex, Tokyo, Japan), serum adiponectin and high sensitivity C-reactive protein (HS-CRP) by latex immunonephelometry (Mitsubishi Chemical Medience, Tokyo, Japan; Siemens Healthcare Diagnostics, Tokyo, Japan), urinary albumin by immunonephelometry (Medical and Biological Laboratories, Nagoya, Japan) and serum total cholesterol, LDL cholesterol, HDL cholesterol, triglyceride, creatinine and urine creatinine by enzymatic methods. Estimated glomerular filtration rate (eGFR) was calculated using the equation proposed by the Japanese Society of Nephrology [25]. β cell function and insulin sensitivity were estimated based on fasting glucose and C-peptide concentrations using the HOMA Calculator, version 2.2.2 [http://www.dtu.ox.ac.uk, accessed June 2012], and expressed as the homeostasis model assessment β cell function (HOMA2-%B) and the homeostasis model assessment insulin sensitivity (HOMA2-%S), respectively. A total of 587 participants with unacceptable levels of plasma glucose (<3 mmol/l or <25 mmol/l) or C-peptide (<0.2 nmol/l or <3.5 nmol/l) were excluded [26]. Albuminuria was defined as urinary albumin excretion ≥30 mg/gCr, and CKD was defined as albuminuria and/or eGFR <60 ml/min/1.73 m^2^[27]. Metabolic syndrome was defined according to the definition of “Harmonizing the Metabolic Syndrome” [28], i.e., the presence of at least two of the following four components: central obesity for Asians (waist circumference ≥90 cm in males and ≥80 cm in females), elevated triglycerides (≥1.69 mmol/l and/or the use of triglyceride-lowering drugs), reduced HDL cholesterol (<1.03 mmo/l in males and <1.29 mmol/l in females) and elevated blood pressure (systolic blood pressure ≥130 mmHg and/or diastolic blood pressure ≥85 mmHg and/or the use of antihypertensive drugs). The presence of depressive symptoms was assessed using the Center for Epidemiologic Studies Depression Scale [29], and subjects who scored more than 16 out of 60 points were defined as having depressive symptoms.

### Statistical analysis

The correlations with dietary fiber intake were assessed by Pearson’s correlation for continuous variables and a logistic regression analysis for categorical variables. The regression coefficients and 95% CIs' were calculated using a multiple regression analysis after multivariate adjustment for potential confounding factors, including age, sex, duration of diabetes, current smoking habits, current drinking habits, total energy intake, fat intake, saturated fatty acid intake, protein intake (only for urinary albumin excretion and eGFR), leisure time physical activity and use of oral hypoglycemic agents or insulin. Due to their skewed distributions, triglyceride, adiponectin and urinary albumin excretion values were log transformed, and the results were expressed as geometric means with 95% CIs. The multivariate-adjusted ORs and 95% CIs for metabolic syndrome and chronic kidney disease were calculated using a multiple logistic regression model. All statistical analyses were performed using the SAS software package version 9.3 (SAS Institute Inc., Cary, NC). Values of P <0.05 were considered to be statistically significant in all of the analyses.

## Results

Table 1 shows the clinical characteristics of the studied participants and correlations with dietary fiber intake. Regarding the source of dietary fiber, vegetables were most frequently eaten, followed by cereals, legumes and beans and fruits, as reported previously in the general population in Japan [16]. Age, fat and protein intakes and leisure time physical activity were positively associated with dietary fiber intake. The proportions of males, current smokers, current drinkers, saturated fatty acid intake and the proportion of participants with depressive symptoms were negatively associated with dietary fiber intake. However, the duration of diabetes and treatment for diabetes were not associated with dietary fiber intake.

**Table 1:** Characteristics of the studied participants and correlations with dietary fiber intake

	**Mean or percentage**	**Correlation coefficient or odds ratio**	**p**
Number	4,399		
Dietary fiber intake (g/1,000 kcal)	7.60 ± 0.03	-	
Fiber from vegetables (%)	47.1 ± 0.2	0.42^*^	<0.0001
Fiber from cereals (%)	22.5 ± 0.2	−0.60^*^	<0.0001
Fiber from legumes and beans (%)	11.8 ± 0.1	0.11^*^	<0.0001
Fiber from fruits (%)	9.5 ± 0.1	0.15^*^	<0.0001
Age (years)	65.4 ± 0.2	0.18^*^	<0.0001
Sex (male, %)	56.7	0.76 [0.74-0.78]^#^	<0.0001
Duration of diabetes (years)	15.5 ± 0.2	0.00^*^	ns
Current smoker (%)	18.3	0.79 [0.76-0.82]^#^	<0.0001
Current drinker (%)	38.8	0.77 [0.74-0.79]^#^	<0.0001
Total energy intake (kcal)	1689 ± 7	−0.12^*^	<0.0001
Fat intake (g/day)	52.4 ± 0.3	0.09^*^	<0.0001
Saturated fatty acid intake (g/day)	12.3 ± 0.1	−0.08^*^	<0.0001
Protein intake (g/day)	67.3 ± 0.4	0.04^*^	0.006
Leisure-time physical activity (met▪hr/week)	11.8 ± 0.2	0.12^*^	<0.0001
Depressive symptoms (%)	8.9	0.95 [0.90-0.99]^#^	0.035
Oral hypoglycemic agents (%)	64.1	0.99 [0.96-1.01]^#^	ns
Insulin therapy (%)	27.1	0.98 [0.95-1.01]^#^	ns

Mean ± SE. *correlation coefficient, ^#^odds ratio with 95% CI.

As shown in Table 2, BMI, waist circumference, fasting plasma glucose (FPG), HbA1c, fasting serum C-peptide, HS-CRP, triglyceride, systolic blood pressure and urinary albumin excretion were significantly and negatively associated with dietary fiber intake after adjusting for age, sex, duration of diabetes, current smoking habits, current drinking habits, total energy intake, fat intake, saturated fatty acid intake, protein intake (only for urinary albumin excretion), leisure time physical activity and use of oral hypoglycemic agents or insulin. The insulin sensitivity index HOMA2%-S, HDL cholesterol and eGFR were significantly and positively associated with dietary fiber intake after multivariate adjustments including protein intake (only for eGFR). The insulin secretion index HOMA2%-B, adiponectin, total cholesterol, LDL cholesterol and diastolic blood pressure were not significantly associated with dietary fiber intake.

**Table 2:** Multiple regression analysis of dietary fiber intake with clinical and laboratory variables

**Variables**	**Mean**	**Regression coefficient**	**p for trend**
Body mass index (kg/m^2^)	23.8 ± 0.06	−0.18 [−0.24,-0.11]	<0.0001
Waist circumference (cm)	85.9 ± 0.2	−0.56 [−0.73,-0.39]	<0.0001
Fasting plasma glucose (mmol/l)	7.73 ± 0.03	−0.049 [−0.084,-0.014]	0.007
HbA1c (%)	7.42 ± 0.02	−0.022 [−0.038,-0.005]	0.009
HbA1c (mmol/mol)	57.6 ± 0.2	−0.24 [−0.42,-0.06]	0.009
Fasting serum C-peptide (nmol/l)	0.402 ± 0.003	−0.009 [−0.013,-0.006]	<0.0001
HOMA2%-B	45.7 ± 0.4	−0.26 [−0.68, 0.17]	ns
HOMA2%-S	106.0 ± 0.6	1.95 [1.27, 2.62]	<0.0001
Adiponectin (μg/ml)*	9.1 [8.9-9.2]	0.006 [−0.003, 0.016]	ns
HS-CRP (mg/l)*	0.50 [0.48-0.52]	−0.067 [−0.090,-0.043]	<0.0001
Total cholesterol (mmol/l)	4.99 ± 0.01	0.009 [−0.005, 0.023]	ns
LDL cholesterol (mmol/l)	2.87 ± 0.01	0.010 [−0.002, 0.022]	ns
HDL cholesterol (mmol/l)	1.47 ± 0.01	0.008 [0.001, 0.014]	0.017
Triglyceride (mmol/l)*	1.22 [1.20-1.24]	−0.013 [−0.022,-0.005]	0.003
Systolic blood pressure (mmHg)	130.7 ± 0.3	−0.35 [−0.64,-0.06]	0.017
Diastolic blood pressure (mmHg)	74.7 ± 0.2	−0.05 [−0.22, 0.13]	ns
Urinary albumin excretion (mg/g)	28.2 [26.8-29.7]	−0.092 [−0.121,-0.063]	<0.0001
eGFR (ml/min/1.73 m^2^)	75.0 ± 0.3	0.34 [0.01, 0.67]	0.042

Mean ± SE. *Geometric means with 95% CI in brackets. HOMA2-%B, homeostasis model assessment β-cell function; HOMA2-%S, homeostasis model assessment insulin sensitivity; HS-CRP, high sensitivity-C reactive protein; eGFR, estimated glomerular filtration rate. The multivariate adjustment included age, sex, duration of diabetes, current smoking habits, current drinking habits, total energy intake, fat intake, saturated fatty acid intake, protein intake (only for urinary albumin excretion and eGFR), leisure time physical activity and use of oral hypoglycemic agents or insulin.

The results of multiple logistic analysis between metabolic syndrome and dietary fiber intake are shown in Table 3. The prevalence of obesity, abdominal obesity, hypertension, hypertriglyceridemia, low HDL cholesterol and metabolic syndrome in the study participants was 31.2%, 47.0%, 73.9%, 29.2%, 18.2% and 54.5%, respectively. Abdominal obesity and hypertension were negatively associated with dietary fiber intake after multivariate adjustments, and further adjustment with obesity did not change the trends. Hypertriglyceridemia and low HDL cholesterol were not associated with dietary fiber intake. Consequently, metabolic syndrome was negatively associated with dietary fiber intake after multivariate adjustment with additional adjustment for obesity.

**Table 3:** Multiple logistic analysis between metabolic syndrome and dietary fiber intake

		**Odds ratio**	**p for trend**
Elevated waist circumference	Model	0.90 [0.87-0.94]	<0.0001
	Model + obesity	0.93 [0.89-0.97]	0.002
Elevated blood pressure	Model	0.93 [0.89-0.97]	0.0002
	Model + obesity	0.94 [0.91-0.98]	0.006
Elevated triglyceride	Model	0.97 [0.93-1.00]	ns
	Model + obesity	0.98 [0.95-1.02]	ns
Low HDL cholesterol	Model	0.97 [0.93-1.01]	ns
	Model + obesity	0.98 [0.93-1.02]	ns
Metabolic syndrome	Model	0.92 [0.89-0.96]	<0.0001
	Model + obesity	0.95 [0.91-0.99]	0.009

Obesity: BMI ≥25.0 kg/m2; Elevated waist circumference, waist circumference ≥90 cm in males and ≥80 cm in females; Elevated blood pressure, systolic blood pressure ≥130 mmHg and/or diastolic blood pressure ≥85 mmHg and/or the use of antihypertensive drugs; Elevated triglyceride, fasting serum triglyceride ≥1.69 mmol/l and/or the use of triglyceride-lowering drugs; Low HDL cholesterol, fasting serum HDL cholesterol <1.03 mmol/l in males and <1.29 mmol/l in females. Metabolic syndrome was defined according to the definition of “Harmonizing the Metabolic Syndrome.” Model, multivariate adjustments with age, sex, duration of diabetes, current smoking habits, current drinking habits, total energy intake, fat intake, saturated fatty acid intake, leisure time physical activity and use of oral hypoglycemic agents or insulin.

The results of multiple logistic analysis between chronic kidney disease and dietary fiber intake are shown in Table 4. The prevalence of albuminuria, low eGFR and chronic kidney disease in the study participants was 38.4%, 21.5% and 46.9%, respectively. Albuminuria, low eGFR and CKD were negatively associated with dietary fiber intake after multivariate adjustments. These trends were not significantly affected by additional adjustment for obesity, hypertension or metabolic syndrome.

**Table 4:** Multiple logistic analysis between chronic kidney disease and dietary fiber intake

		**Odds ratio**	**p for trend**
Albuminuria ≥30 mg/g	Model	0.92 [0.88-0.95]	<0.0001
	Model + obesity	0.93 [0.89-0.96]	<0.0001
	Model + elevated blood pressure	0.93 [0.89-0.96]	<0.0001
	Model + metabolic syndrome	0.93 [0.89-0.96]	<0.0001
eGFR < 60 ml/min/1.73 m^2^	Model	0.94 [0.90-0.98]	0.006
	Model + obesity	0.95 [0.90-0.99]	0.015
	Model + elevated blood pressure	0.95 [0.91-0.99]	0.019
	Model + metabolic syndrome	0.95 [0.91-0.99]	0.027
Chronic kidney disease	Model	0.93 [0.90-0.96]	<0.0001
	Model + obesity	0.94 [0.90-0.97]	0.0005
	Model + elevated blood pressure	0.94 [0.91-0.97]	0.0009
	Model + metabolic syndrome	0.94 [0.91-0.98]	0.0011

Obesity: BMI ≥25.0 kg/m2; Elevated waist circumference, waist circumference ≥90 cm in males and ≥80 cm in females; Elevated blood pressure, systolic blood pressure ≥130 mmHg and/or diastolic blood pressure ≥85 mmHg and/or the use of antihypertensive drugs; Elevated triglyceride, fasting serum triglyceride ≥1.69 mmol/l and/or the use of triglyceride-lowering drugs; Low HDL cholesterol, fasting serum HDL cholesterol <1.03 mmol/l in males and <1.29 mmol/l in females. Metabolic syndrome was defined according to the definition of “Harmonizing the Metabolic Syndrome.” Model, multivariate adjustments with age, sex, duration of diabetes, current smoking habits, current drinking habits, total energy intake, fat intake, saturated fatty acid intake, protein intake, leisure time physical activity and use of oral hypoglycemic agents or insulin.

## Discussion

The present study demonstrated that dietary fiber intake was associated with better glycemic control and more favorable CVD risk factors including abdominal obesity, hypertension and metabolic syndrome, along with enhanced insulin sensitivity and reduced HS-CRP after adjusting for confounding factors. Furthermore, the proportion of participants with CKD negatively associated with dietary fiber intake, even after adjusting for obesity, hypertension or metabolic syndrome. To the best of our knowledge, there are few epidemiological studies showing associations of dietary fiber intake with glycemia and CVD risk factors in Asia, where the epidemic of type 2 diabetes is rapidly becoming a serious medical and socioeconomic issue.

A recent systematic review of the literature reported that adding fiber supplements in moderate amounts (4–19 g) to daily diet achieved little improvement in glycemic control or CVD risk factors [9]. On the other hand, another meta-analysis [2] of intervention trials using high fiber diet (mean increase in fiber 18.3 g/d) in type 2 diabetic patients revealed that FPG and HbA1c were modestly lowered by 0.83 mmol/l and 0.26%, respectively, compared with a placebo. In the present study, both FPG and HbA1c negatively associated with dietary fiber intake (Table 2). In addition, the insulin sensitivity index HOMA2%-S and HS-CRP were associated with dietary fiber intake and the association remained statistically significant after the additional adjustment for BMI (regression coefficient 1.34 [0.71, 1.97], -0.048 [−0.070,-0.025], respectively). Although the effects of dietary fiber on insulin sensitivity have not been studied in type 2 diabetic patients, dietary fiber enhances insulin sensitivity in hepatic and peripheral tissues in insulin-resistant obese subjects [3-5].

It was recently reported that the consumption of high fiber diet for four weeks enhanced insulin secretion in nondiabetic overweight subjects [30]. Dietary fiber may activate incretin secretion due to short-chain fatty acid production induced by the fermentation of dietary fiber [31], although, in one study, it took one year for high fiber diet to enhance glucagon-like peptide-1 secretion in healthy subjects [32]. In the present study, the insulin secretion index HOMA2%-B was not associated with dietary fiber intake, suggesting that it is unlikely that insulin secretion induced by increased dietary fiber intake contributes to improving hyperglycemia.

In general, dietary fiber favorably affects CVD risk factors, including LDL cholesterol [33] and components of metabolic syndrome [34,35]. In type 2 diabetic patients, a recent review reported that high fiber diet failed to affect the lipid levels in four out of eight randomized controlled studies [9]. In the present study, total cholesterol and LDL cholesterol were not associated with dietary fiber intake. However, HDL cholesterol and triglyceride were significantly associated with dietary fiber intake. Dietary fiber exerts blood pressure-lowering effects [36,37], and recently, Jenkins et al. [38] reported that high fiber and low glycemic index diet with legumes reduced blood pressure compared with wheat fiber diet in type 2 diabetic patients. In the present study, systolic blood pressure and hypertension were negatively associated with dietary fiber intake. Enhanced insulin sensitivity may contribute to the blood pressure-lowering effects of dietary fiber. As a result, the prevalence of metabolic syndrome was significantly associated with dietary fiber intake. Reduced fiber intake, particularly at breakfast, was found to be associated with metabolic syndrome in Brazilian type 2 diabetic patients [10,39], although the authors did not report which component of metabolic syndrome was associated with low fiber intake. The present study demonstrated that dietary fiber intake was associated with reduced prevalence of abdominal obesity and hypertension of metabolic syndrome phenotypes independent of obesity. A reduction in abdominal obesity induced by increased dietary fiber intake has been reported in both intervention [40,41] and epidemiological studies [42]. However, the direct effects of dietary fiber on visceral adipose tissue remain to be elucidated.

CKD is an established CVD risk factor. The present study demonstrated the association between dietary fiber intake and lower prevalence of CKD (Table 4). Due to the cross-sectional nature of the study, preventing hyperkalemia in the advanced stage of CKD may limit the consumption of fresh fruits and green vegetables. Indeed, in this study, the proportion of participants with eGFR < 30 ml/min/1.73 m^2^ was negatively associated with dietary fiber intake (odds ratio 0.83 [0.76-0.91]). However, excluding participants with eGFR < 30 ml/min/1.73 m^2^ (n = 115) did not change the results (odds ratio 0.94 [0.91-0.97]). CVD risk factors, such as obesity, hypertension and metabolic syndrome, may contribute to the development and progression of CKD. However, adjusting for each CVD risk factor did not change the significant association between dietary fiber intake and CKD (Table 4). Although the mechanisms of action of dietary fiber in the kidneys are unknown, high dietary fiber intake is associated with a lower level of systemic micro-inflammation in both nondiabetic and diabetic patients [8,12], as shown in the present study (Table 2). The anti-inflammatory actions of dietary fiber may be related to reduced prevalence of CKD. Recently, a large follow-up study showed that increased dietary fiber intake was associated with reduced mortality in CKD patients [43]. In this context, dietary fiber appears to be promising non-pharmacological treatment for CKD.

The strength of the present study includes a relatively large sample size of type 2 diabetic patients consuming foods different from Western diet [7]. A staple food in the Japanese diet is white rice, which has lower dietary fiber than whole grains. The amount of daily fiber intake in Japan declined from 20.5 g/d to 15 g/d after World War II [44] to a level that is lower than that observed in the US and UK [7]. The main source of dietary fiber of Japanese people is vegetables including seaweed, a typical Japanese food, followed by cereals, legumes and fruits [16]. The present study showed that the dietary fiber present in Japanese foods exerts beneficial effects on glycemia and CVD risk factors, thus suggesting that the usefulness of increased dietary fiber intake may extend beyond certain ethnic foods. Another strength of the study is that confounding factors included fat and saturated fatty acid intakes and physical activity, since high dietary fiber intake is often associated with healthy lifestyle, making it difficult to isolate fiber effects from general healthy lifestyle [14]. However, some limitations should be discussed. First, the use of a self-administered food frequency dietary assessment questionnaire BDHQ is subject to measurement error in dietary intake, and actual dietary habits may not be obtained. However, the ability to rank dietary fiber using the BDHQ has been reasonably verified [22]. Second, study participants who visit diabetologists regularly may be better educated about self-management of diabetes with respect to diet than the general population. However, the daily fiber intake of the study participants was similar to that of the general population in Japan (15 g/d). Third, since multiple outcomes were involved in the present study, multiple testing may induce false results. Finally, we cannot prove cause-and-effect relationships due to the cross-sectional design of our study, and there may be other confounding factors in addition to those evaluated in the present study.

## Conclusion

We demonstrated that increased dietary fiber intake was associated with better glycemic control and more favorable CVD risk factors including hypertension, metabolic syndrome and CKD, along with improvements in insulin sensitivity and micro-inflammation, in Japanese type 2 diabetic patients after adjusting for confounding factors. Although the recommended amount of dietary fiber in the general population is <19 g/d for males and <17 g/d for females in Japan [45] and <38 g/d for males and <25 g/d for females in the US [46], diabetic patients should be encouraged to consume more dietary fiber in daily life according to the ethnic foods.

## Abbreviations

BDHQ: Brief-type self-administered diet history questionnaire; BMI: Body mass index; CKD: Chronic kidney disease; CVD: Cardiovascular disease; eGFR: Estimated glomerular filtration rate; FPG: Fasting plasma glucose; HS-CRP: High sensitivity C-reactive protein; HOMA2-%B: Homeostasis model assessment β cell function; HOMA2-%S: Chomeostasis model assessment insulin sensitivity.

## Competing interests

The authors declare that they have no competing interests.

## Authors’ contributions

HF and MI were responsible for the study concept and design. HF and MI conducted the analyses, and TO, SO, HI, YK, YI, TJ, YH, KU, SS, UN and TK helped with interpreting the data and contributed to the discussion. HF and MI drafted the manuscript. All authors participated in revising the manuscript critically and approved the final version.
